# EBF1 is expressed in pericytes and contributes to pericyte cell commitment

**DOI:** 10.1007/s00418-021-02015-7

**Published:** 2021-07-16

**Authors:** Francesca Pagani, Elisa Tratta, Patrizia Dell’Era, Manuela Cominelli, Pietro Luigi Poliani

**Affiliations:** 1 Pathology Unit, Department of Molecular and Translational Medicine, University of Brescia Medical School, P.le Spedali Civili 1, 25125 Brescia, BS Italy; 2 Cellular Fate Reprogramming Unit, Department of Molecular and Translational Medicine, University of Brescia, Brescia, BS Italy

**Keywords:** Pericytes, Angiogenesis, Cell fate commitment, Mesenchymal stem cells

## Abstract

Early B-cell factor-1 (EBF1) is a transcription factor with an important role in cell lineage specification and commitment during the early stage of cell maturation. Originally described during B-cell maturation, EBF1 was subsequently identified as a crucial molecule for proper cell fate commitment of mesenchymal stem cells into adipocytes, osteoblasts and muscle cells. In vessels, EBF1 expression and function have never been documented. Our data indicate that EBF1 is highly expressed in peri-endothelial cells in both tumor vessels and in physiological conditions. Immunohistochemistry, quantitative reverse transcription polymerase chain reaction (RT-qPCR) and fluorescence-activated cell sorting (FACS) analysis suggest that EBF1-expressing peri-endothelial cells represent *bona fide* pericytes and selectively express well-recognized markers employed in the identification of the pericyte phenotype (SMA, PDGFRβ, CD146, NG2). This observation was also confirmed in vitro in human placenta-derived pericytes and in human brain vascular pericytes (HBVP). Of note, in accord with the key role of EBF1 in the cell lineage commitment of mesenchymal stem cells, EBF1-silenced HBVP cells showed a significant reduction in PDGFRβ and CD146, but not CD90, a marker mostly associated with a prominent mesenchymal phenotype. Moreover, the expression levels of VEGF, angiopoietin-1, NG2 and TGF-β, cytokines produced by pericytes during angiogenesis and linked to their differentiation and activation, were also significantly reduced. Overall, the data suggest a functional role of EBF1 in the cell fate commitment toward the pericyte phenotype.

**Supplementary Information:**

The online version contains supplementary material available at 10.1007/s00418-021-02015-7.

## Introduction

Early B-cell factor 1 (EBF1) represents a highly evolutionarily conserved DNA-binding transcription factor with an atypical zinc-finger and helix-loop-helix motif that belongs to a family of four genes (*ebf1*, *ebf2*, *ebf3*, *ebf4*) (Liao 2009) with an important role in cellular differentiation during the development of different tissues (Medina et al. 2004; Busslinger et al. 2000; Liberg et al. 2002). Specifically, in humans, EBF1 has been found to be involved in different developmental pathways, including B-cell differentiation (Lukin et al. 2008), neurogenesis (Garel et al. 1999; Garcia-Dominguez et al. 2003; Acosta et al. 2016), osteogenesis (Kieslinger et al. 2005) and adipogenesis (Akerblad et al. 2002; Jimenez et al. 2007). It is also reported that EBF transcriptional factors play a role in tumorigenesis (Mullighan et al. 2007). Recently, we reported that EBF3 is highly expressed in medulloblastoma, a malignant embryonal brain tumor, and acts as a major master regulator of neuronal differentiation (Corno et al. 2012). During this study we performed immunohistochemistry using a pan-EBF antibody and we observed strong and specific immunoreactivity in newly formed tumor vessels. The vascular unit mainly consists of endothelial cells, pericytes, vascular smooth muscle cells (vSMCs) and fibroblasts (Diaz-Flores et al. 2009). We first investigated which of the four EBF family members was expressed in the vascular unit and we identified pericytes as EBF1-expressing cells. Pericytes are contractile cells surrounding the endothelial layer of small blood vessels (Armulik et al. 2011, 2010). Although their function is not completely clarified, pericytes have an important role in different processes including the regulation of blood flow (Kutcher et al. 2009) and the maintenance of the blood–brain barrier (Armulik et al. 2010). In particular, pericytes exert an important function in vascular development, interacting with endothelial cells through a dynamic and complex paracrine cross-talk. Of note, pericytes are frequently confused with mesenchymal stem cells (MSCs) or other mesenchymal-derived cells around vessels, such as vSMCs. Nevertheless, it is well documented that pericytes are ubiquitously present in microvessels, particularly capillaries, while vSMCs are typically located around the arterioles and precapillary arterioles (Armulik et al. 2011). To date, no specific markers are available for unequivocally distinguishing pericytes from MSCs (Raza et al. 2010; Blocki et al. 2013). Indeed, MSCs and pericytes share the same embryological origin, and pericytes themselves are thought to derive from mesodermal progenitors (Armulik et al. 2011). Since EBFs have been described as major regulators of MSC differentiation (Kiviranta et al. 2013), we also investigated whether EBF expression allows for the selective discrimination of pericytes from MSCs.

## Materials and methods

### Immunohistochemistry and immunocytochemistry

Immunohistochemistry (IHC) and immunocytochemistry (ICC) were performed on formalin-fixed, paraffin-embedded (FFPE) tissue samples, including tissue microarray, and cell blocks retrieved from the pathology service of Spedali Civili of Brescia. The primary antibodies used are listed in Table 1. All the antibodies are commercially available and validated for use in FFPE tissue samples. Antibody specificity was tested on the recommended positive controls according to the manufacturers’ data sheets. The validation of the EGFRvIII antibody was reported previously (Cominelli et al. 2015). EBF1 expression in tumors was semi-quantitatively scored based on both percentage and intensity of immunoreactive cells with a combined cumulative score ranging from 0 to 6. Cell block preparation and immunostaining procedures were performed as described in the Supplementary Material. Images were acquired with a Nikon DS-Ri2 camera (4908 × 3264 full-pixel) mounted on a Nikon Eclipse 50i microscope equipped with Nikon Plan lenses (x10/0.25; x20/0.40; x40/0.65; x100/1.25) using NIS-Elements 4.3 imaging software (Nikon Corporation).

**Table 1:** Primary antibodies used in the study

Mouse monoclonal anti-EBF3, clone 8D6 (not specific; pan-EBF)	Abnova	H00253738-M05
Rabbit polyclonal anti-EBF1 (IHC)	Merck Millipore	AB10523
Rabbit polyclonal anti-EBF1 (WB)	MyBioSource	MBS2027769
Rabbit polyclonal anti-EBF2	Merck Millipore	AB10524
Rabbit polyclonal anti-EBF3	Merck Millipore	AB10525
Rabbit polyclonal anti-EBF4	Biorbyt	orb183290
Mouse monoclonal anti-CD31, clone 1A10	Leica Novocastra	PA0250
Mouse monoclonal anti-CD34, clone QBEnd/10	Leica Novocastra	PA0212
Rabbit polyclonal anti-FVIII	Thermo Scientific	RB-281-A
Rabbit monoclonal anti-PDGFRb, clone 28E1	Cell Signaling Technology	3169
Mouse monoclonal anti-SMA	Biocare Medical	CM 001 A
Mouse monoclonal anti-CD146, clone N1238	Leica Novocastra	NCL-CD146
Rabbit polyclonal anti-NG2	Chemicon	AB5320
Mouse monoclonal anti-calponin clone CALP	Agilent Dako	M3556
Rabbit polyclonal anti-CD90	Abnova	PAB30354
Goat polyclonal anti-TIE2	Santa Cruz Biotech	sc-31266
Mouse monoclonal anti-CD68, clone KP1	Leica Novocastra	M0814
Mouse monoclonal anti-CD163, clone 10D6	Thermo Scientific	MS-1103-S1
Mouse monoclonal anti-GFAP, clone 6F2	Agilent Dako	M0761
Mouse monoclonal anti-IDH1, clone H09	Dianova	DIA H09
Rabbit polyclonal anti-EGFRvIII*	GenScript USA Inc	Custom-made against peptide LEEKKGNYVVTDHC
Mouse monoclonal anti-actin, clone AC-40 (WB)	Sigma-Aldrich	A4700

*Kindly provided by Dr. G. Finocchiaro, Neurological Institute “Besta”, Milan, Italy

### Cell isolation, maintenance and transfection

Pericytes were isolated from human placentas provided by the birthing room at Spedali Civili of Brescia in accordance with the protocol approved by the ethics committee (protocol no. 1842) and following procedures described within the Supplementary Material. Human umbilical vein endothelial cells (HUVECs) were isolated from umbilical veins as described previously (Pagani et al. 2018). Human brain vascular pericytes (HBVP) and human cerebral microvascular endothelial cells (HCMEC) were obtained from ScienCell. Adipose tissue MSCs (MSC-AT) were isolated from human adipose tissue as reported previously (De Luca et al. 2013). U87 were kindly provided by Prof. Ronca, University of Brescia. All primary cultures were used at early passages (II-VI). For transfection, 25 × 10^3^ HBVP cells/cm^2^ were seeded in 12-well or 6-well plates and incubated with EBF1 gene-specific or control siRNAs (1 nM final concentration) (Origene, ID-SR301317). A universal scrambled negative control siRNA duplex (SCR) was used as negative control.

### Immunophenotypical analysis and immunofluorescence

Immunophenotypical analysis was performed by fluorescence-activated flow cytometry (FACSCalibur, BD Bioscience). Single cell suspensions (100,000 cells/300 µL) were incubated 25 min with monoclonal CD45-FITC, CD146-PE, CD90-APC, CD31-Pe-Cy7 or isotype-matched IgG control antibodies (BD Biosciences). Data were analyzed with FlowJo software. Immunofluorescence was carried out on cells seeded on coverslips and fixed in 4% paraformaldehyde (PFA) in phosphate-buffered saline (PBS) solution. Cells were incubated with primary antibodies overnight at 4 °C following 1 h incubation with secondary Alexa Fluor 488 anti-mouse IgG (Thermo Fisher) and fluorescein isothiocyanate (FITC)-conjugated goat anti-rabbit IgG (DAKO). Nuclei were counterstained with DAPI. Images were acquired with a Cohu high-performance CCD camera (4912–5010/000) mounted on a Nikon Eclipse 90i microscope equipped with Nikon Plan lenses (×100/1.25) using the Genikon imaging system 3.7.8 (Nikon Corporation) (DAPI filter: 49000_Nikon DIH-90i; FITC filter: 49011_Nikon DIH-90i).

### Western blotting

Equal amounts of protein extracts (40 µg) obtained from HBVPs 24 h after transfection were separated by 10% sodium dodecyl sulfate polyacrylamide gel electrophoresis (SDS-PAGE) and transferred to polyvinylidene difluoride (PVDF) membrane following incubation with rabbit polyclonal anti-EBF1, rabbit monoclonal anti-PDGFRβ, clone 28E1 and mouse monoclonal anti-actin, clone AC-40 (Table 1). Membranes were then incubated with the specific horseradish peroxidase-conjugated secondary antibodies and immunocomplexes revealed using a chemiluminescence detection kit (Euroclone) with the Odyssey^®^ imaging system (LiCor Biosciences). Densitometric analysis was performed with ImageJ software.

### Evaluation of Ki-67 expression

After transfection, the medium was replaced with the following media: basal pericyte medium (Sciencell) without supplementation; pericyte medium with the addition of Pericyte Growth Supplement (PGS) and 2% fetal bovine serum (FBS); pericyte medium with 50 µL/mL of medium obtained from U87 glioblastoma cells cultured for 48 h under hypoxic conditions. After 24 h, cells were collected for RNA extraction, and quantitative reverse transcription polymerase chain reaction (RT-qPCR) for Ki-67 was performed as described in the following section.

### RNA isolation and RT-qPCR

Total RNA from cell cultures was isolated using TRIzol reagent (Invitrogen) following the manufacturer’s instructions. One microgram of total RNA was reverse-transcribed using the iScript cDNA synthesis kit for first-strand cDNA synthesis (Bio-Rad). RT-qPCR was performed with iTaq™ Universal SYBR^®^ Green Supermix (Bio-Rad) (Supplementary Table 1). Glyceraldehyde-3-phosphate dehydrogenase (GAPDH) mRNA was used as endogenous reference for relative quantification.

### Statistical analysis

The *t*-test and one-way analysis of variance with Dunnett's post hoc test were applied to compare the different groups. Results were analyzed by GraphPad Prism 6.01 software, and values of *p* < 0.05 were considered statistically significant. Data were presented as mean ± standard deviation of at least three independent experiments.

## Results

### EBF1 is expressed in vessels in both neoplastic and non-neoplastic conditions

**Fig. 1:**
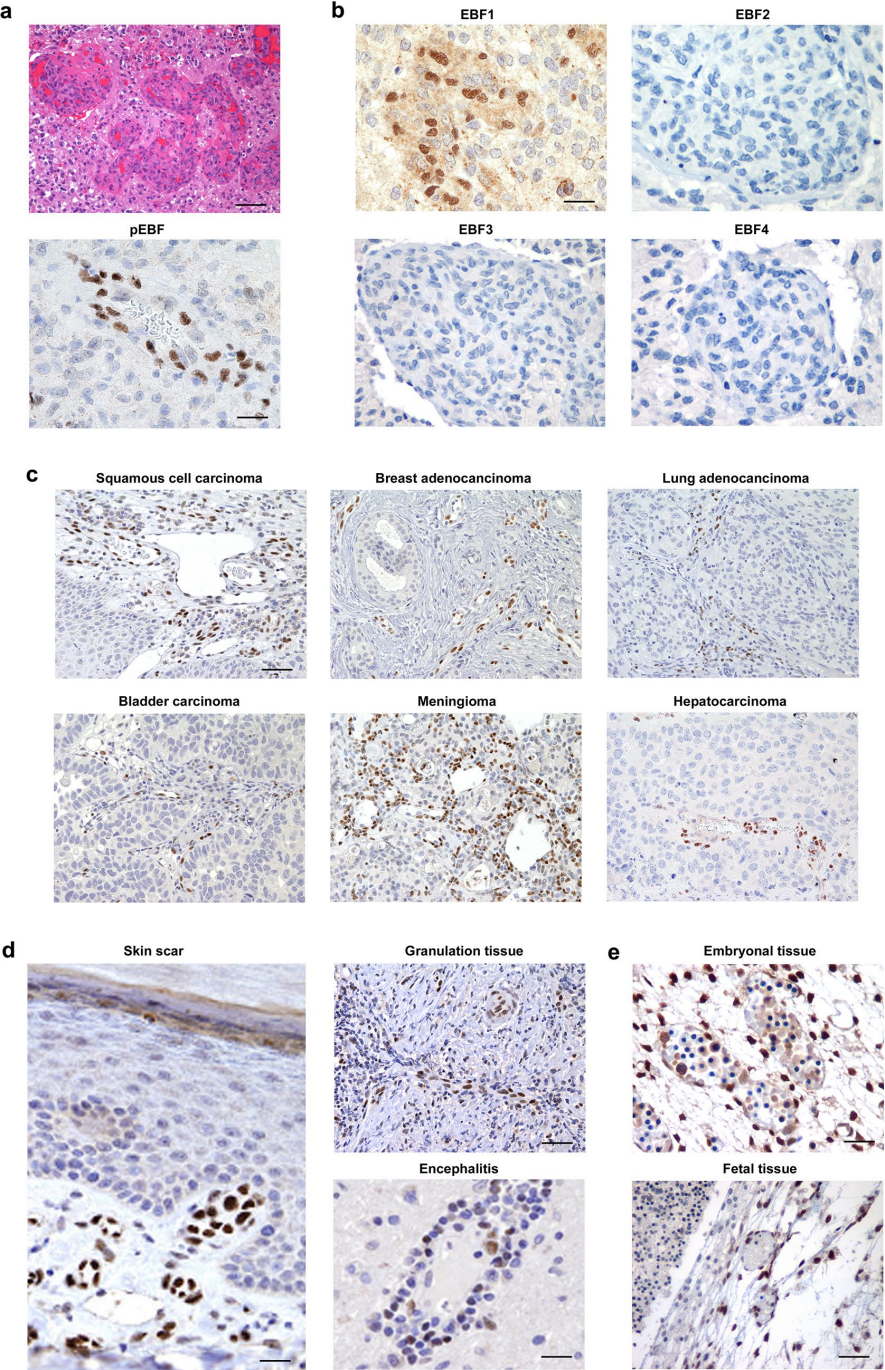
EBF1 is expressed in a cell population within the vessel wall in both neoplastic and non-neoplastic conditions. **a** Using an anti-pan-EBF antibody, glomeruloid vascular proliferation in glioblastoma (upper image, H&E staining, ×20 original magnification) shows immunoreactivity for EBFs (lower image, anti-pan-EBF immunostain, ×40 original magnification). **b** When specific antibodies for all the different EBF family members (EBF1–4) were employed, only EBF1 showed positive immunostaining (all images are ×40 original magnification). **c** Representative sections from different tumor samples show that newly formed tumor vessels selectively express EBF1 independently of their histotype, suggesting that EBF1 expression is a common feature of tumor vessels (all images are ×10 original magnification). **d** EBF1 is also expressed in the walls of small vessels in physiological and pathological non-neoplastic conditions, i.e. surgical scars (left image; ×40 original magnification), granulation tissue (upper right image; ×20 original magnification) and inflammatory lesions such as encephalitis (lower right image; ×40 original magnification). **e** EBF1 is strongly expressed in the vessel wall in embryonic (upper image, ×40 original magnification) and fetal tissue (lower image, ×20 original magnification). From panel (**c**–**e**) all immunostaining was performed with a specific antibody for EBF1. Scale bars: ×10, ×20 and ×40 original magnification, corresponding respectively to 200 μm, 100 μm and 50 μm

As reported in a previous study (Corno et al. 2012), we performed IHC for EBF3 using a pan-EBF antibody recognizing all four EBF family members. We used glioblastoma, a highly malignant glial tumor known to lack EBF expression, as negative control. Interestingly, we observed strong and specific immunoreactivity in newly formed tumor vessels, while neoplastic cells were negative (Fig. 1a). Since the anti-pan-EBF antibody recognizes all four EBF proteins, we employed specific antibodies for the different molecules, EBF1–4, and observed that the glomeruloid glioblastoma vessels selectively expressed EBF1 (Fig. 1b). However, we wondered whether expression of EBF1 was restricted to glioblastoma vessels or was indeed a common feature of tumor vessels. To test this hypothesis we searched for EBF1 expression using tissue microarrays and representative sections of tumor samples of different histotypes (Table 2). Interestingly, we observed selective EBF1 expression within tumor vessels of all the samples analyzed, independently of their histotype (Fig. 1c). To assess whether EBF1 is also expressed in vessels in physiological and/or pathological non-neoplastic conditions, we investigated EBF1 expression on representative sections from different normal tissues (brain, skin, thyroid, bladder, pancreas, kidney, lung, cervix, colon, stomach, omentum) and in representative non-neoplastic conditions where angiogenesis is active (surgical scar, granulation tissue, inflammatory diseases, including encephalitis). In normal samples from physiological conditions, EBF1 was expressed only sporadically in cells within the small vessels (Supplementary Fig. 1a). However, in vessels from a skin surgical scar, we found a higher number of EBF1-expressing cells (Fig. 1d), as well as in vessels from non-neoplastic pathological conditions, such as granulation tissue and inflammation (Fig. 1d). We also investigated EBF1 expression in samples from both embryonic and fetal tissues, conditions in which angiogenesis is active. As expected, we found strong EBF1 expression in cells within newly formed vessels (Fig. 1e). In summary, data indicate sustained and specific expression of EBF1 in a cell population within the small vessel wall in both neoplastic and non-neoplastic conditions.

**Table 2:** List of the tumor samples from different tissues and histotype subjected to immunohistochemical analysis for the expression of EBF1

Tumor	Number (TMA)	Number (entire sections)
Lung	10	7
Head-neck	10	/
Pancreas	10	3
Thyroid	10	3
Colon	10	5
Kidney	10	3
Bladder	10	2
Breast	10	8
Skin	10	6
Stomach	10	4
Liver	10	8
Oropharynx	–	5
Leiomyoma/leiomyosarcoma	–	6
Meningioma	–	4
Ovary/cervix/endometrium	16	10

*TMA* Tissue Microarrays

### EBF1 immunoreactive cells are pericytes

**Fig. 2:**
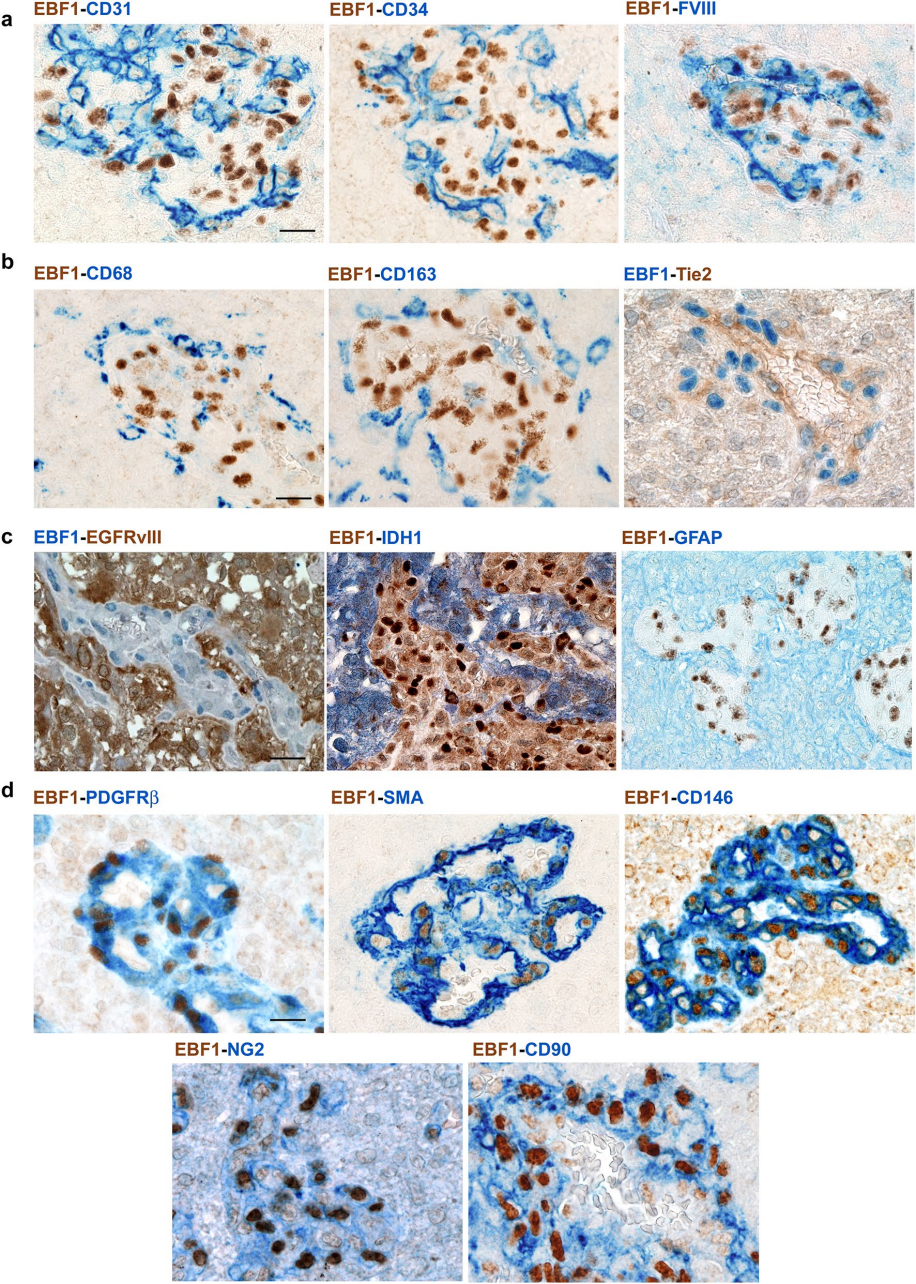
EBF1-expressing cells are pericytes. Double immunostaining combining the specific antibody for EBF1 and lineage-specific markers was applied for glioblastoma samples with prominent glomeruloid vascular proliferation in order to identify the phenotype of EBF1-expressing cells. Double immunostaining using endothelial markers CD31, CD34, FVIII showed no double-positive cells (**a**), and the same was found for monocyte/macrophage markers (CD68, CD163, Tie-2) (**b**) and for the histological hallmarks of tumor-derived glioma cells (GFAP, EGFRvIII, IDH1-R132H) (**c**). In contrast, double immunostaining using the most widely recognized mesenchymal/pericyte markers (PDGFRβ, SMA, CD146, NG2 and CD90) (**d**) revealed the pericyte phenotype of EBF1-expressing cells. Chromogen used to identify immunoreactivity is either brown or blue according to the corresponding label, as indicated. All images are ×40 original magnification; scale bar corresponds to 50 μm

**Fig. 3:**
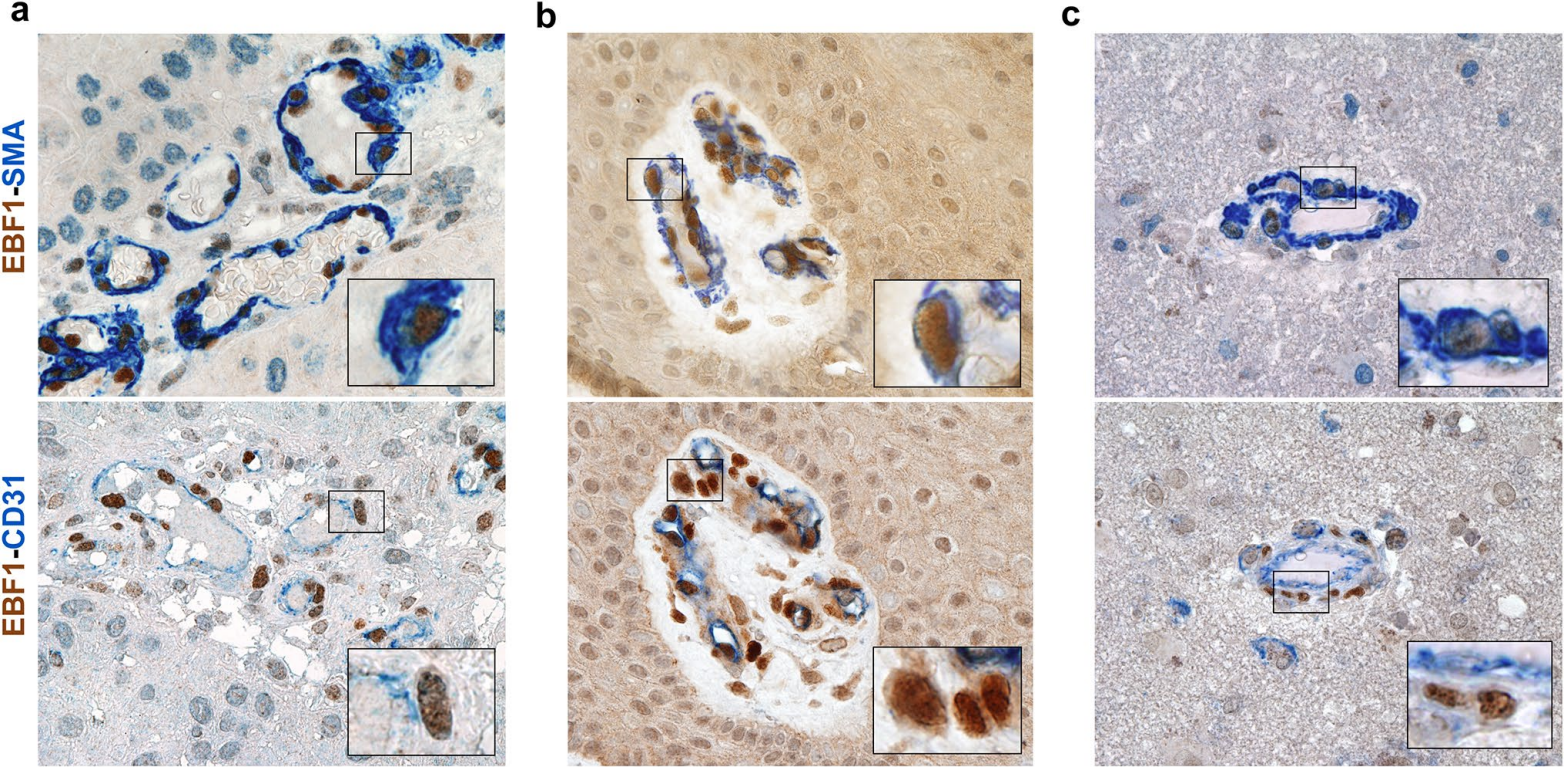
EBF1-expressing cells are pericytes. Double immunostaining for EBF1 combined with either pericyte (SMA) or endothelial (CD31) lineage-specific markers on representative tissue sections from other neoplastic lesions (squamous cell carcinoma; left upper and lower images), physiological conditions (skin scar; middle upper and lower images) and inflammatory conditions (encephalitis; right upper and lower images) confirmed the pericyte phenotype of EBF1-expressing cells. Remarkably, the double staining with the endothelial markers clearly highlights the peri-endothelial distribution of the EBF1-expressing cells. In double immunostaining, EBF1 shows nuclear staining, while all the other markers showed cytoplasmic or membrane staining. Chromogen used to identify immunoreactivity is either brown or blue according to the corresponding label, as indicated. Higher magnification of the co-localized areas. All images are ×40 original magnification; scale bar corresponds to 50 μm. Insets are photographic enlargement of a representative field, as indicated

Different cell types are present within the vascular wall, including endothelial cells (Mae et al. 2011), monocytes/macrophages (Yamamoto et al. 2017; Yamazaki et al. 2017), tumor cells (Cheng et al. 2013; Ricci-Vitiani et al. 2010) and pericytes (Armulik et al. 2011). In order to detect the phenotype of EBF1-expressing cells, we thus performed double immunostaining of glioblastoma samples using a specific antibody for EBF1 combined with lineage-specific markers. We first hypothesized that EBF1-expressing cells may belong to the endothelial cell lineage. However, double immunostaining showed that EBF1-expressing cells did not express the endothelial markers CD31, CD34, or FVIII (Fig. 2a). In addition, EBF1-expressing cells did not express monocyte/macrophage markers CD68, CD163 or Tie-2, recognized markers of tumor-associated macrophages with pro-angiogenic properties (De Palma et al. 2005) (Fig. 2b). Since recent data have shown that glioblastoma cancer stem cells can generate cells contributing to vessel formation (Cheng et al. 2013; Ricci-Vitiani et al. 2010), we performed double immunostaining using antibodies that selectively recognize the mutated proteins EGFRvIII and IDH1-R132H, histological hallmarks of tumor-derived glioma cells. As shown, EBF1-expressing cells did not express EGFRvIII or IDH1-R132H, indicating that they are not of neoplastic origin. Moreover, EBF1+ cells did not express GFAP, a well-recognized marker of glioblastoma (Fig. 2c). Finally, we performed double immunostaining using the most widely recognized mesenchymal/pericyte markers (SMA, PDGFRβ, CD146, NG2, CD90). Immunostaining revealed the pericyte phenotype of EBF1-expressing cells that were all positive for pericyte markers, including PDGFRβ, SMA, CD146, NG2 and CD90 (Fig. 2d). We further performed double immunostaining for EBF1 combined with either pericyte (SMA) or endothelial (CD31) lineage-specific markers on tissue sections from other pathological and physiological conditions, as previously shown. Data confirmed the pericyte phenotype of EBF1-expressing cells, located in a peri-endothelial space (Fig. 3 and Supplementary Fig. 1b). Overall, the combination of both phenotype and topographical location within the wall of the small vessels suggests that the EBF1-expressing cells are *bona fide* pericytes.

### EBF1 is expressed in pericytes isolated from both human placenta and brain

**Fig. 4:**
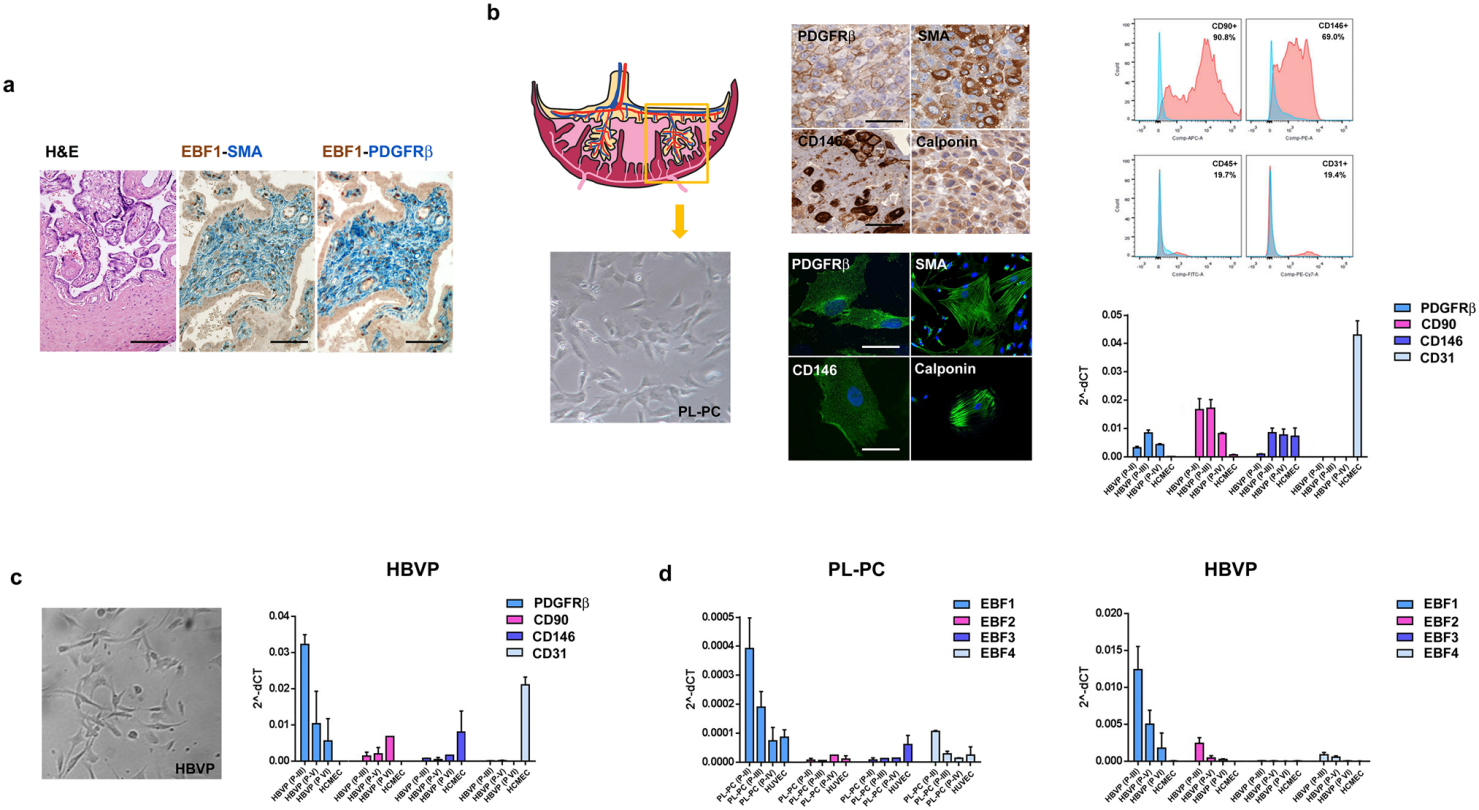
EBF1 is expressed in pericytes isolated from both human placenta and brain. **a** Representative section from human placenta (left image, H&E staining; ×20 original magnification) shows that EBF1-positive cells within the vessel wall have a pericyte phenotype, as previously described. Double immunostaining for EBF1 (brown nuclear) and SMA, PDGFRβ (blue cytoplasmic); ×20 original magnification. **b** Placental-derived pericytes (PL-PC; left images, ×40 original magnification) were phenotypically characterized by immunocytochemistry on cell blocks (upper middle panels; ×40 original magnification) and immunofluorescence on cultured cells (lower middle panels; ×100 original magnification) using pericyte markers (PDGFRβ, SMA, CD146, calponin), confirming their pericyte phenotype. PL-PCs at passage II (P-II) were also analyzed by flow cytometry using specific antibodies for CD90-APC, CD146-PE, CD45-FITC and CD31-Pe-Cy7 (red). PL-PCs were positive for the pericyte/mesenchymal markers CD90 and CD146 and negative for CD45 (hematopoietic marker) and CD31 (endothelial marker). The negative control is also shown (no antibodies, light blue) (upper right images). RT-qPCR analysis (lower right image) shows that the pericyte phenotype is maintained in culture at least until P-IV, as indicated by the expression of PDGFRβ, CD90 and CD146 and the negativity for CD31. The endothelial counterpart (HUVECs used as control) shows a higher level of CD31 expression, as expected. Of note, CD146 was also detected in HUVECs, in accord with reported data (Du et al. 2015). **c** Commercially available HBVPs were used as a second pericyte cell line of different embryological origin (neuroectoderm) as compared to PL-PCs (mesoderm) (left image, ×40 original magnification). Their pericyte phenotype was confirmed by RT-qPCR (right image). As expected, HBVPs expressed PDGFRβ, CD90 and CD146, whereas HCMECs, the endothelial counterpart, expressed CD31. HBVPs maintained the pericyte phenotype in culture at least until P-VI even with a progressive decrease in PDGFRβ expression. **d** The expression of the EBF family members was assayed by RT-qPCR in both PL-PCs and HBVPs and their endothelial counterparts. As expected, both PL-PCs (left histogram) and HBVPs (right histogram) expressed high levels of EBF1, while the expression of the other EBF family members was very low or barely detected. Of note, the absolute expression level of EBF1 in HBVPs was more than 20-fold higher than that of PL-PCs, probably related to their different embryological origin. Scale bars: ×20, ×40 and ×100 original magnifications, corresponding respectively to 100 μm, 50 μm and 20 μm

To confirm the expression of EBF1 in pericytes, we used an in vitro model based on isolation of pericytes from human placenta, a highly vascularized tissue easily available and particularly enriched in pericytes (Bjarnegård et al. 2004). Firstly, we investigated the expression of EBF1 in placental tissue samples. As expected, we observed the presence of EBF1-expressing cells in placental vascular structures, confirming their pericyte phenotype (SMA+, PDGFRβ+) by double immunostaining (Fig. 4a). Thus, we isolated human placental pericytes by a custom-modified protocol as described. Placental-derived pericytes (PL-PC) were phenotypically characterized by immunocytochemistry, immunofluorescence, flow cytometry and RT-qPCR. HUVECs isolated from the umbilical vein were used as the endothelial counterpart. Analysis confirmed that PL-PCs were *bona fide* pericytes. We confirmed the expression of pericyte markers in PL-PCs by both immunocytochemistry on cell blocks and immunofluorescence on cell culture (CD146, PDGFRβ, calponin, SMA) (Fig. 4b, left panels). PL-PCs at passage II (P-II) analyzed by flow cytometry were positive for the pericyte/mesenchymal markers CD90 and CD146 and negative for CD45 (hematopoietic marker) and CD31 (endothelial marker) (Fig. 4b, upper right panel). We did not analyze early passages (P-0 and P-I) due to the presence of a low percentage of contaminating cells of other cell types (e.g. fibroblasts, hematopoietic and endothelial cells) that were negatively selected during the following passages in a medium enriched by a pericyte growth supplement. Finally, we also confirmed the pericyte phenotype of PL-PCs by RT-qPCR. As shown, the pericyte phenotype is maintained in culture at least until P-IV. Pericytes, as compared with HUVECs, express high levels of PDGFRβ and CD90, while they do not express the endothelial marker CD31 (Fig. 4b, lower right panel). Interestingly, the putative pericyte marker CD146 was also detected with a comparable level of expression in HUVECs. Indeed, it is reported in the scientific literature that CD146 is also a marker of endothelial lineage (Du et al. 2015). CNS pericytes are considered to derive from the neural crest (Korn et al. 2002; Kurz et al. 2004; Etchevers et al. 2001), while the large majority of pericytes are considered to derive from the mesothelium (Que et al. 2008; Armulik et al. 2011). For this reason, since we used glioblastoma as a model to characterize the phenotype of EBF1-expressing cells, we also investigated EBF1 expression in HBVPs. Even though HBVPs were commercially available, we confirmed the pericyte nature of these cells by both flow cytometry (not shown) and RT-qPCR. As expected, HBVPs showed a pericyte phenotype. Of note, RT-qPCR showed high levels of PDGFRβ and CD90, and was negative for CD31. HCMECs were used as the endothelial counterpart. In contrast to PL-PCs, HBVPs expressed lower levels of CD146 as compared to HCMECs, which expressed a comparable level of CD146 as HUVECs (Fig. 4c). HBVPs retained the pericyte phenotype in culture at least until P-VI even with a progressive decrease in PDGFRβ expression. For this reason, we did not use HBVPs for our analyses after P-VI. We then investigated by RT-qPCR the expression of EBF family members in PL-PCs and HBVPs and their endothelial counterparts. As expected, both PL-PCs and HBVPs expressed high levels of EBF1 while the expression of the other EBF family members was very low or barely detected (Fig. 4d). Of note, the absolute level of expression of EBF1 in HBVPs was more than 20-fold higher than that of PL-PCs. Overall, these data confirmed that pericytes express EBF1.

### EBF expression distinguishes pericytes from MSCs

**Fig. 5:**
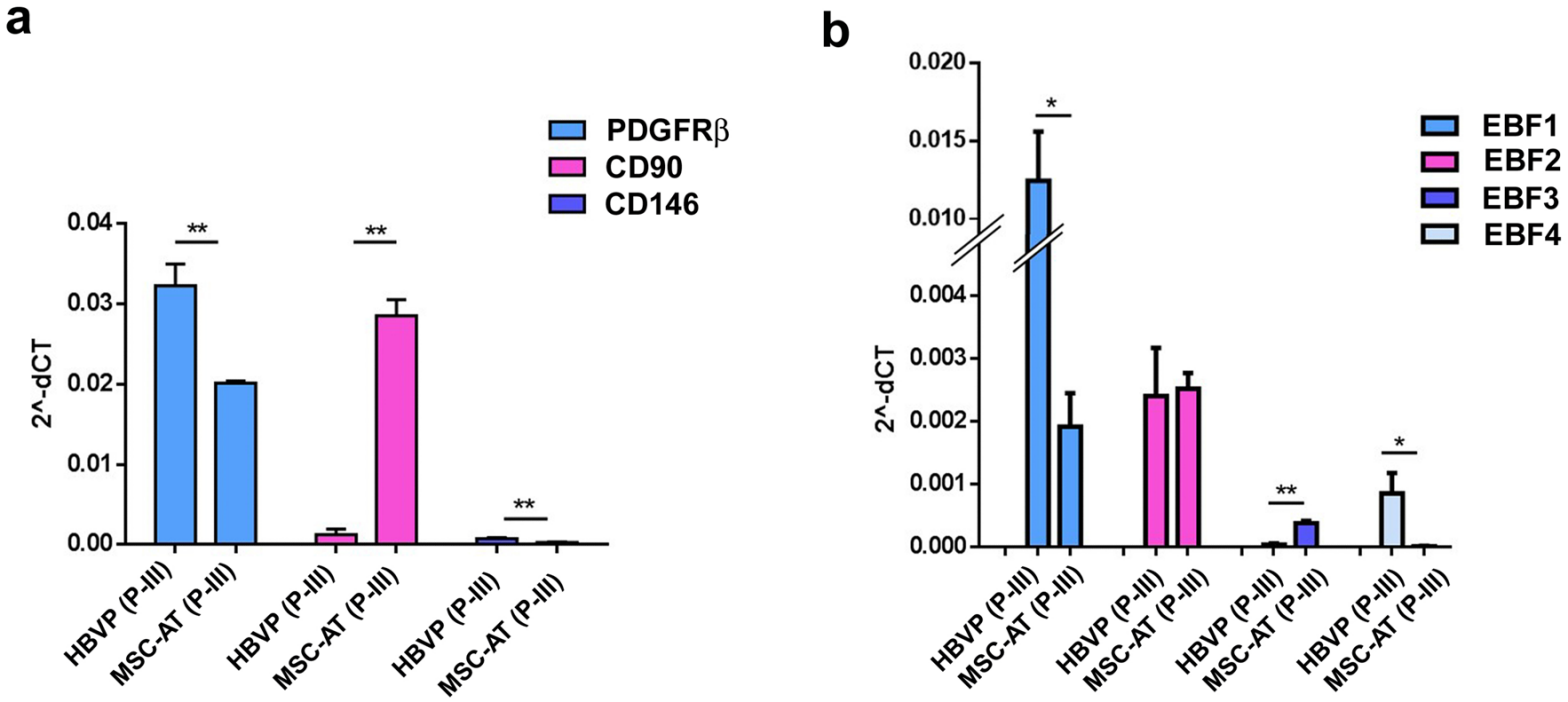
EBF expression allows pericytes to be distinguished from MSCs. **a** MSCs isolated from human adipose tissue (MSC-AT) showed, as expected, significantly lower levels of PDGFRβ as compared to HBVPs (*p* < 0.01), with high levels of CD90 that conversely was barely detected in HBVPs (*p* < 0.01). Of note, CD146 was expressed, albeit at low levels, only in HBVPs (*p* < 0.01). **b** The expression levels of the EBF family members in MSC-AT and HBVPs showed that EBF1 was expressed also in MSC-AT, albeit at a significant lower level as compared to HBVPs (*p* < 0.05). While EBF2 was expressed at a comparable level in both MSC-AT and HBVPs, EBF3 was detected only in MSC-AT, albeit at a very low level (*p* < 0.01). Of note, EBF4 was detected only in HBVPs, although at a very low level (*p* < 0.05). These data suggest that the EBF expression profile allows MSC-AT to be distinguished from pericytes. *p < 0.05, **p < 0.01

Given the close correlation between pericytes and MSCs (Crisan et al. 2008), we investigated the expression of EBF family members also in MSCs. To this end, we used MSCs isolated from human adipose tissue (MSC-AT). Both MSC-AT and HBVPs were assayed at P-III. Phenotypical characterization of both MSC-AT and HBVPs highlights significantly higher expression of PDGFRβ in HBVPs as compared to MSC-AT, as well as for CD146, even at a lower level of expression. In contrast, the expression of CD90 was significantly higher in MSC-AT while it was barely detectable in HBVPs (Fig. 5a). We then compared the expression levels of EBF family members in both MSC-AT and HBVPs. Data showed that also MSC-AT express EBF1, albeit at a significant lower level as compared to HBVPs. Of note, while both MSC-AT and HBVPs express low levels of EBF2, EBF3 was detected only in MSC-AT, albeit at a very low level. Conversely, EBF4 was detected only in HBVPs, also at a very low level (Fig. 5b). Data suggest that the EBF expression profile could be a useful tool in pericyte/MSC characterization and that the combination of EBF expression and cell lineage markers enables MSC-AT to be differentiated from pericytes with high confidence.

### EBF1 is involved in the pericyte phenotype cell commitment

**Fig. 6:**
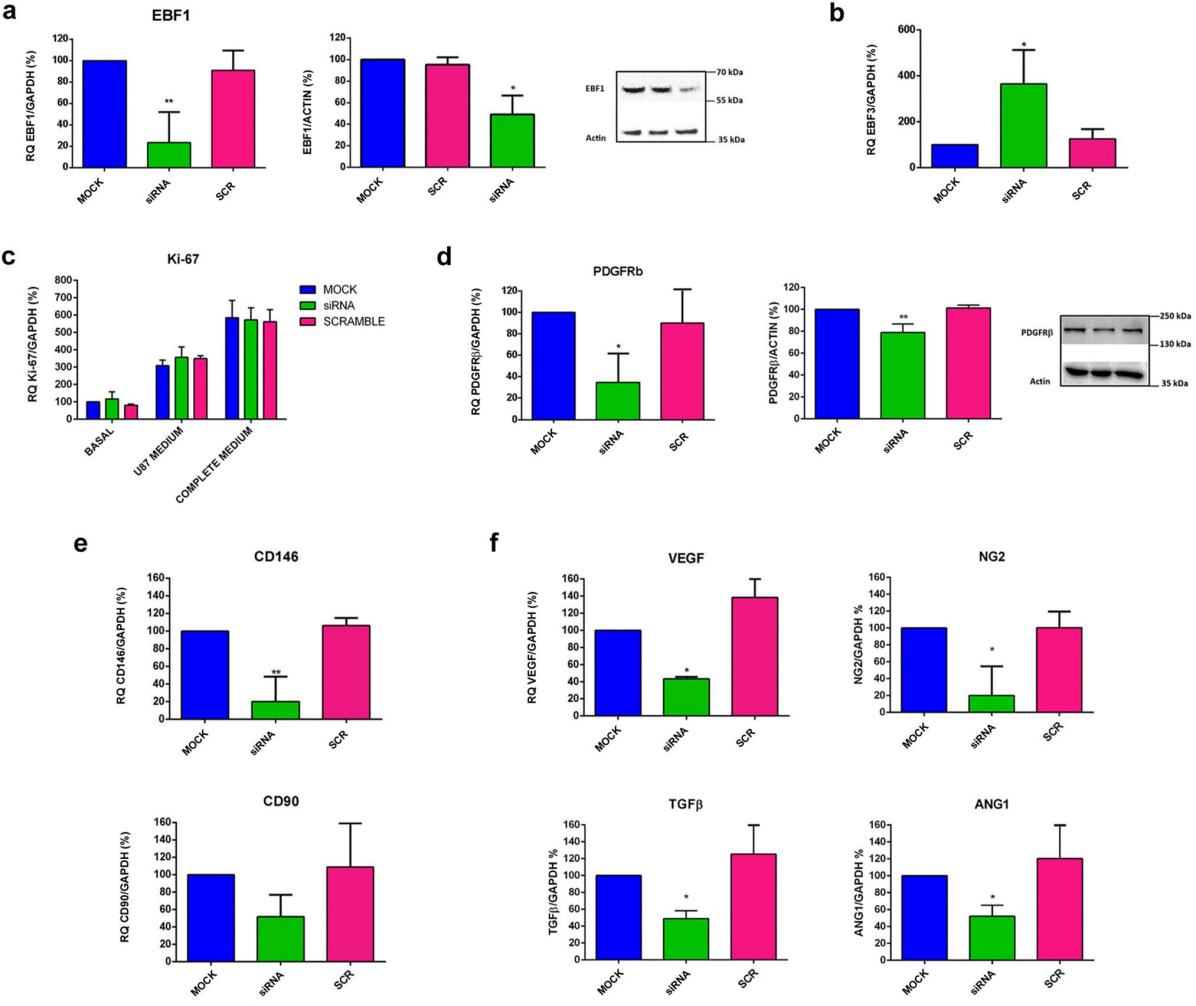
EBF1 plays a key role in the pericyte phenotype cell commitment. **a** Significant downregulation of EBF1 mRNA levels (about 75% decrease; *p* < 0.01) was obtained in HBVPs by means of siRNA technology, measured by RT-qPCR 24 h after transfection (upper histogram). Immunoblotting performed with protein extracts confirmed the reduction in the synthesis of EBF1 protein (about 50% decrease; *p* < 0.05). The representative image on the right shows the corresponding EBF1 band at 66 kDa. **b** Histogram shows that after EBF1 silencing, the expression of EBF3, a marker linked to the MSC phenotype, is significantly increased (*p* < 0.05). **c** Silencing of EBF1 does not affect cell proliferation as assessed by the evaluation of the nuclear antigen Ki-67 expression by RT-qPCR, 24 h after seeding cells with or without the addition of different proliferative stimuli, such as the medium from U87 glioblastoma cells cultured under hypoxic conditions or the complete culture medium containing PGS and 10% FBS. **d** We then investigated whether EBF1 silencing affects the phenotype of the pericytes. Indeed, the histograms show a significant reduction in the main pericyte markers PDGFRβ (*p* < 0.05) at both the transcriptional (*p* < 0.05) and protein (*p* < 0.01) levels. **e** Additionally, we found a significant reduction in the pericyte marker CD146 (*p* < 0.01). The expression levels of CD90, a marker linked to a mesenchymal phenotype, did not show a significant reduction. **f** Of note, the expression levels of VEGF, Ang-1, NG2 and TGF-β, cytokines produced by pericytes during different phases of angiogenesis are significantly reduced in EBF1-silenced HBVPs (*p* < 0.05). Overall, data confirmed a functional role of EBF1 in pericyte cell fate commitment and functionality. *p < 0.05, **p < 0.01. CTRL (control); siRNA (EBF1-silenced); SCR (scramble, control siRNA); Basal (basal conditions); Hypoxic (hypoxic conditions)

In order to investigate the functional role of EBF1 in pericytes, we exposed HBVPs (Supplementary Fig. 2) and PL-PCs (not shown) to different culture conditions, i.e. hypoxia, nutrient starvation and medium obtained from U87 glioblastoma cells cultured under hypoxic and normoxic conditions. Data show that EBF1 expression did not change at 24, 48, or 72 h after exposure. We then performed functional studies downregulating EBF1 expression in HBVPs by means of siRNA technology. We selected the best-performing siRNA out of three different molecules and we identified the concentration of 1 nM as that providing the best ratio between silencing efficiency and absence of off target effects (not shown). We obtained significant downregulation of EBF1 mRNA levels (approximately 75%) as measured by RT-qPCR 24 h after transfection. Immunoblotting performed with protein extracts collected 24 h after transfection confirmed the reduction in the synthesis of EBF1 protein, with a 50% decrease of the band corresponding to the protein (66 kDa) (Fig. 6a). We did not observe any change in the level of EBF2 or EBF4 mRNAs in these cells, and transfection with an unrelated control siRNA (SCR) resulted in no modification of the expression of any of the EBF genes (not shown). Interestingly, we found a significant increase in the expression of EBF3 in cells silenced for EBF1 (Fig. 6b). To investigate whether the lack of EBF1 affects cell proliferation and survival, the same number of HBVP cells was plated and cultured at basal conditions or in proliferative conditions, i.e. with the complete culture medium containing pericyte growth supplement and FBS or, as an alternative, the medium obtained from U87 glioblastoma cells cultured under hypoxic conditions. After 24 h, cells were collected and we evaluated the expression of the nuclear antigen Ki-67 as a marker of proliferation. We could not find any differences in Ki-67 expression levels in silenced cells compared to mock control or SCR-treated cells, or at basal or under the different proliferative stimuli (Fig. 6c). Additionally, cells were counted after 48 h and we could not find any differences in the proliferation rate of silenced cells compared to mock control or SCR-treated cells, or at basal conditions or with addition of the U87 glioblastoma hypoxia-conditioned medium (Supplementary Fig. 3). We did not detect any sign of cell death in any samples. To verify the consequences of EBF1 silencing on the cell fate commitment, we analyzed the expression level of pericyte markers, as previously described. Of note, we found a significant reduction in the main pericyte marker, PDGFRβ, at transcriptional level. Immunoblotting performed with protein extracts collected 24 h after transfection confirmed the reduction in the synthesis of the protein, with a 35% statistically significant reduction of the band corresponding to the protein (190 kDa) (Fig. 6d). We also found a significant reduction in the pericyte marker CD146 at transcriptional level, whereas we did not find a significant reduction in the expression levels of CD90, a marker linked mainly to a prominent mesenchymal phenotype (Fig. 6e). We then analyzed the expression levels of remarkable angiogenic molecules produced by pericytes during angiogenesis and strictly related to pericyte functionality, i.e. VEGF, angiopoietin-1, NG2 and TGF-β1. In the early phases of angiogenesis, VEGF (Darland et al. 2003) and NG2 are secreted for the purpose of promoting endothelial cell survival and motility (Fukushi et al. 2004). Pericytes can also secrete TGF-β, a multifunctional cytokine essential for the formation of vessels (Walshe et al. 2009), and contribute to blood–brain barrier integrity through the stabilization of the actin filaments in endothelial cells (Dohgu et al. 2005). Angiopoietin-1 is produced by pericytes in the later stages of angiogenesis, with a role in vessel maturation (Sundberg et al. 2002). Interestingly, the expression levels of all these angiogenic molecules were significantly reduced in HBVPs silenced for EBF1 (Fig. 6f). Overall, the data suggest a functional role of EBF1 in pericyte cell differentiation that is also reflected in cell functionality.

## Discussion

Pericytes are an essential component of the vascular unit, with an important functional role in angiogenesis, vessel stability and homeostasis. As mentioned above, no specific markers are available to date that can unequivocally distinguish pericytes from other components of the vascular unit, particularly from MSCs. Herein, we have identified a *bona-fide* pericyte marker: EBF1. Our data indicate that EBF1-expressing cells selectively display well-recognized markers employed in the identification of the pericyte phenotype (SMA, PDGFRβ, CD146, NG2) and are negative for the endothelial markers CD31, CD34 and FVIII. Up to now, there are no reported data concerning the expression of EBF1 in pericytes. Of note, in a previous study, using laser-capture microdissection and transcriptional analysis, the authors identified CD248 and PDGFRβ, both related to the pericyte phenotype, within upregulated molecules in glioma-associated vessels in comparison to normal vessels (Dong et al. 2005). According to our observations, in the same manuscript, the authors also identified EBF1 as a significantly upregulated gene in microdissected glioma vessels surrounding palisading cells, a typical feature of glioblastoma where angiogenesis is particularly active. In addition, EBF1 was found to be expressed in normal human brain vasculature with respect to whole brain, as assayed by RNA sequencing analysis of microvessel preparations isolated from human brain samples by laser-capture microdissection (2.51-fold change; *p*-value = 3.82E−05) (Song et al. 2020). These data were derived from available lists of over-expressed genes. However, EBF1 expression in vessels has never been further investigated. Several reports have shown that, in agreement with their close ontogenetic relationship, pericytes and MSCs show a similar phenotype, and none of the putative pericyte markers are strictly specific and, at variable levels, are also expressed in MSCs. Based on these observations, some reports speculate that there are no major differences between pericytes and MSCs, both being considered cells with stem cell properties (Crisan et al. 2008; Caplan 2008; Murray et al. 2014). Indeed, our data suggest that, although derived from a common progenitor, pericytes have their own properties and represent a different cell type. Interestingly, as shown, the combined expression of different EBFs represents a robust specific signature that makes it possible to discriminate between pericytes, which strongly express EBF1, and to some extent also EBF4, and MSCs, which express higher levels of EBF3 and lower levels of EBF1, and are consistently negative for EBF4. Of note, we have also shown that expression of EBF3 is significantly increased in EBF1-silenced HBVP cells, in line with the evidence that EBF3 is related to the mesenchymal phenotype. Moreover, in a recent work, the authors performed transcriptome analysis to compare human umbilical cord-derived MSC and HBVPs. Interestingly, EBF1 was one of the top five out of 43 genes upregulated more than tenfold in HBVPs compared with MSCs (23.831-fold change; *p* value = 1.85E−14) (Guijarro-Muñoz et al. 2014). These reports further support our data indicating that EBF1 is expressed in peri-endothelial cells within small vessels whose phenotype and topographical location can be ascribed to pericytes. In addition, a recent study has shown that EBF1 is expressed in podocytes during nephrogenic development and is maintained into adulthood (Fretz et al. 2014). Podocytes are considered specialized pericyte-like cells covering the glomerular capillary endothelial cell layer of the Bowman's capsule in the kidney. In this work, the authors demonstrated that in ebf1−/− mice, the reduced glomerular maturation was mainly related to a failure of podocytes to properly upregulate VEGF and promote epithelial and mesangial recruitment. It is well known that VEGF is produced by pericytes during angiogenesis (Darland et al. 2003). These data support the observation that lack of EBF1 expression negatively affects maturation of podocytes, with consequent decreased VEGF production, as we also observed in pericytes silenced for EBF1. We have also shown that MSCs express lower levels of PDGFRβ and CD146 as compared to HBVPs, but show high levels of CD90, a recognized mesenchymal marker that is indeed expressed at a significantly lower level in pericytes. Interestingly, supporting this observation, we have shown that enforced silencing of EBF1 expression in HBVPs negatively affects the pericyte phenotype, as indicated by significant downregulation of PDGFRβ and CD146. Moreover, EBF1 silencing affects the production of angiogenic molecules produced by pericytes during angiogenesis and associated with their differentiation and activation, such as VEGF (Du et al. 2015), NG2 (Fukushi et al. 2004), TGF-β (Walshe et al. 2009) and angiopoietin-1 (Dohgu et al. 2005; Sundberg et al. 2002). It is well known that the activating transcription factor 5 (ATF5) regulates cell differentiation, survival and apoptosis in a wide range of different cells (Mason et al. 2005; Sears and Angelastro 2017), including MSCs (Leong et al. 2007, 2012). It has also been reported that EBF1 binds to the ATF5 promoter and regulates ATF5 transcription (Wei et al. 2010). Of note, BCL-2 is one of the downstream target of ATF5 that mediates the cell cycle exit (Dluzen et al. 2011). We can speculate that the EBF1-ATF5-BCL2 axis may promote the cell cycle exit and differentiation of committed progenitors, including their differentiation toward the pericyte cell lineage. Experimental evidence suggests that pericytes from different organs have different origins (Paul et al. 2012). It was hypothesized that in coelomic organs, pericytes derive from mesodermal progenitors (Armulik et al. 2011; Que et al. 2008; Kawaguchi et al. 2007), while in the head and neck region, including the central nervous system (CNS), a subset of pericytes derives from differentiation of neural crest progenitors (Korn et al. 2002; Kurz et al. 2004; Yamamoto et al. 2017). As described, we studied the phenotype and EBF1 expression in pericytes derived from both mesodermal (PL-PC) and neuroectodermal (HBVP) progenitors. EBF1 was expressed in both cell populations, according to their pericyte phenotype. Of note, PL-PCs express lower levels of EBF1 along with lower levels of PDGFRβ as compared to HBVPs. Conversely, CD90 was found to be highly expressed in PL-PCs, suggesting a closer relationship with their mesodermal origin. In our glioblastoma model, hypoxia plays a key role in promoting tumor angiogenesis and induction of the typical glomeruloid vascular proliferation (Dong et al. 2005). Immunostaining performed on human glioblastoma samples showed that the constitutive elements of these glomeruloid structures are represented by EBF1-expressing pericytes, as reported. It is well described that hypoxic stimuli from the tumor microenvironment support activation of neoangiogenesis through different processes, including pericyte proliferation and recruitment. As described, HBVPs exposed to conditioned medium obtained from glioblastoma cells cultured under hypoxic conditions show increased proliferation. Interestingly, we did not find any difference between EBF1-silenced and control cells in term of cell number, indicating that EBF1 is not involved in cell proliferation, in line with previous observations that EBF1 is indeed involved in cell fate commitment of pericyte progenitors. Since tumor angiogenesis has a key role during the development and maintenance of neoplasms, and pericytes represent a pivotal element contributing to tumor angiogenesis, the identification of a functional role of EBF1 provides important information about mechanisms involved in the control and development of tumor angiogenesis, with implications for the development of therapeutic interventions. In summary, our study allowed us to identify EBF1 as a novel marker of pericytes with a putative functional role in the cell commitment toward the pericyte phenotype, an issue never investigated so far.

## Supplementary Information

Below is the link to the electronic supplementary material.

Supplementary file1 (DOCX 855 KB)

## Data Availability

All data generated or analyzed during this study are included in this published article (and its supplementary information files).
