# Supplementary material for: EBF1 is expressed in pericytes and contributes to pericyte cell commitment
Source: Histochem Cell Biol. 2021 Jul 16;156(4):333–47. doi: 10.1007/s00418-021-02015-7 (PMC8550016; doi:10.1007/s00418-021-02015-7)
Supplement: Supplementary file 1 — Supplementary file1 (DOCX 855 KB) [file 418_2021_2015_MOESM1_ESM.docx]

**EBF1 IS EXPRESSED IN PERICYTES AND CONTRIBUTES TO PERICYTE CELL COMMITMENT**

Francesca Pagani^1^, Elisa Tratta^1^, Patrizia Dell’Era^2^, Manuela Cominelli^1^, Pietro Luigi Poliani^1^

Author affiliations: ^1^Pathology Unit and ^2^Cellular Fate Reprogramming Unit, Department of Molecular and Translational Medicine, University of Brescia.

**SUPPLEMENTARY MATERIALS AND METHODS**

**Immunohistochemistry and Immunocytochemistry**

Immunostains were performed on both formalin-fixed paraffin embedded (FFPE) tissue samples and cell blocks. The latter were prepared by resuspending 6 million cells in physiological solution (NaCl 0,9%) and plasma and thromboplastin were added dropwise until the formation of a clotted sphere. The sphere was immediately formalin fixed and paraffin embedded. 2 µm sections were cut both from paraffin embedded cells blocks and tissue samples, provided by Pathological Department of Spedali Civili of Brescia and analyzed as single specimen or in tissue micro-array. Briefly, sections were de-waxed, rehydrated, treated for antigen retrieval, and incubated in 10% serum blocking solution before incubation with primary antibodies at room temperature for 1 hour or at 4°C for 18 hours (Supplementary Table 1). The reaction was revealed by using Novolink^TM^ Polymer Detection System (Novocastra^TM^) or Dako EnVision System-HRP Labelled Polymer anti-mouse or anti rabbit (Dako Cytomation) followed by DAB and slides counterstained with Hematoxylin. For double immunohistochemistry, after completing the first immune reaction, the second one was revealed by using MACH4 Universal AP Polymer kit (Biocare Medical) followed by Ferangi Blue^TM^ Chromogen kit (Biocare Medical) and nuclei were counterstained with Methyl Green. EBF1 expression in pericytic-derived tumours was semi-quantitatively scored on representative tumour regions based on both percentage [score ranges: 0 (0-5%), 1 (6-29%), 2 (30-69%), 3 (≥70%)] and intensity (score ranges: 0, no expression; 1, weak; 2, moderate; 3, high) of immunoreactive neoplastic cells with a combined cumulative score ranging from 0 to 6. Images were acquired with Nikon DS-Ri2 camera (4908x3264 full-pixel) mounted on Nikon Eclipse 50i microscope using NIS-Elements AnalySIS imaging imaging software 4.3 (Nikon Corporation). Scale bars: 10x, 20x, 40x and 100x original magnifications correspond respectively to 200µm, 100µm, 50µm and 5µm.

**Cells isolation, maintenance and transfection**

Pericytes were isolated from human placentas provided by the birthing room at Spedali Civili of Brescia, according to the ethical requirements of the institutional committee on human experimentation, in accordance with the protocol approved by the Ethics Committee of Brescia (prot. n.1842). After mechanical and enzymatic dissociation of specimens with 0,1 U/mL of collagenase type I and 0,8 U/mL of dispase I (Sigma Aldrich) and treatment with Red Blood Cell Lysing Buffer Hybri-Max (Sigma) to remove red blood cells, single-cell suspensions were plated and cultured in Pericyte Medium (Sciencell) supplemented with Pericyte Growth Supplement (PGS) (Sciencell), 20% fetal bovine serum (FBS), penicillin 100 U/ml, and streptomicin 100 µg/ml (BioSera, Nuaille, France)at 37°C, 5% CO_2_, H_2_O saturated atmosphere. HBVP were obtained from ScienCell Research Laboratories and cultured in Pericyte Medium (Sciencell) supplemented with Pericyte Growth Supplement (PGS) (Sciencell), 2% fetal bovine serum (FBS), penicillin 100 U/ml, and streptomicin 100 µg/ml (BioSera, Nuaille, France). HUVECs were previously isolated from umbilical veins according to an established protocol[[1](#_ENREF_1)],in accordance with the protocol approved by the Ethics Committee of Brescia (prot. n.1842). Human Cerebral Microvascular Endothelial Cells (HCMEC) were obtained from ScienCell Research Laboratories. Adipose Derived Mesenchymal Stem Cells were isolated from human adipose tissue as previously shown[[2](#_ENREF_2)]. U87 were kindly provided by prof. R. Ronca, University of Brescia. All the primary cultures were used at early passages (II-VI). Cells were maintained at 37˚C under 5% CO_2,_ H_2_O saturated atmosphere. For the evaluation of EBF1 expression, HBVP and PL-PC were cultured in different conditions: Pericyte Medium (Sciencell) with PGS and 2% or 20% FBS respectively (control); Pericyte Medium without PGS or FBS (starvation); Pericyte Medium without PGS or FBS with the addition of 50µl/ml of medium obtained from U87 Glioblastoma cells cultured for 48 hours under hypoxic or normoxic conditions. Hypoxia for pericytes and U87 cells was obtained by maintaining culture plates at 37°C under a constant flow of a hypoxic gas mixture (Biogroup), composed of 90% N2, 5% O2 and 5% CO2, for 15 minutes. Then plates were sealed and incubated at 37°C for the desired time of hypoxia. After 24, 48 and 72 hours, pericytes were collected in TRIzol for RNA extraction. After 48 hours, U87 conditioned medium was collected, filtered and used as a proliferative stimulus. For EBF1 silencing, one set of three different 27-mer siRNAs specific duplexes was obtained from Origene (ID SR301317). Out of them, the siRNA SR312773A was the most efficient and hence selected for all the described experiments (data not shown). We used a universal scrambled negative control siRNA duplex (SCR) from the same company as a negative control in all transfection experiments. For transfection, 25x10^3 HBVP cells/cm^2 were seeded in 12-wells or 6-wells plates. After 16 h they were incubated with the specific and control siRNAs (1nM final concentration) and 3 μl or 7.5 μl of ScreenFect® siRNA Transfection Reagent (Screenfect GmbH). After 5 hours of incubation, the transfection medium was replaced by Pericyte Medium (Sciencell) supplemented with 2% fetal bovine serum (FBS). Mock-treated cells received only the transfection reagent without the siRNA. After the transfection, cells were collected and analyzed as described in the following sections.

**Cell viability/proliferation.**After transfection, medium was replaced with basal Pericyte Medium (Sciencell) without PGS or FBS, with or without 50µl/ml of medium obtained from U87 Glioblastoma cells cultured for 48 hours under hypoxic conditions. After 48h, cells were detached with trypsin 0.1%/EDTA, stained with trypan blue to check for vitality, and counted.

**Supplementary Table 1.** Primers used in this study

| EBF1 | for 5’CCCTCTTATCTGGAACATGCTACT  rev 5’CAACTCACTCCAGACCAGCA |
| --- | --- |
| EBF2 | for 5’AAGACCAACAACGGCACTCA  rev 5’TTCGCAGCATCGACTACACA |
| EBF3 | for 5’GCATGAAACAGAAGAGCGCC  rev 5’GCCCAGACATAGCTTGCAGT |
| EBF4 | for 5’CTGGCACGAGCACATTTTGA  rev 5’GCTCTGTCCGCAGTCCATTG |
| PDGFRβ | for 5’CAGTGGGGAACAGACAGTCC  rev 5’AGCTCACGTGGACACCTTTT |
| CD90 | for 5’TCCCGAACCAACTTCACCAG  rev 5’ACCAGTTTGTCTCTGAGCACT |
| CD31 | for 5’TCCACATCAGCCCCACCGGA  rev 5’TGGGCCACAATCGCCTTGTCC |
| CD146 | for 5’GTCTGCGCCTTCTTGCTCG  rev 5’CTCCTTGTGGACAGAAAACCAG |
| VEGF | for 5’ GGGCCTCCGAAACCATGAAC  rev 5’ TCCATGAACTTCACCACTTCGT |
| Ki67 | for 5’GAAAGAGTGGCAACCTGCCTTC  rev 5’GCACCAAGTTTTACTACATCTGCC |
| Angiopoietin-1 | for 5’CAATGGGGGAGGTTGGACTGTA  rev 5’GAGGGATTTCCAAAACCCATTTTAT |
| NG2 | for 5’TTGTCCTGATGGCTAATGCCT  rev 5’TGGGCTGCTCGATGGTGTA |
| TGFβ-1 | for 5’GCAGCACGTGGAGCTGTA  rev 5’CAGCCGGTTGCTGAGGTA |
| GAPDH | for 5’ GAAGGTCGGAGTCAACGGATT  rev 5’ TGACGGTGCCATGGAATTTG |


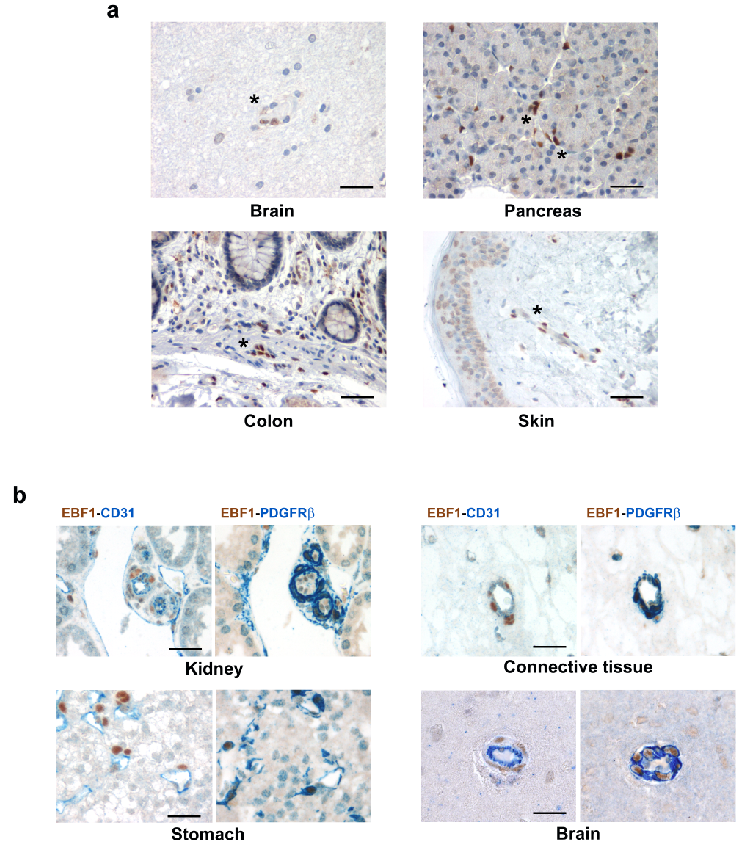


**Supplementary Figure 1. EBF1 expression in representative sections from different normal tissues.** (a) EBF1 expression in vessels from representative normal tissues (brain, pancreas, colon and skin) (upper panels, 40x original magnification; lower panels, 20x original magnification). (b) Double immunostains confirm the pericytic phenotype of EBF1 peri-endothelial expressing cells that all were negative for CD31 and positive for PDGFRβ. Representative tissue sections from normal samples are shown (kidney, connective tissue, stomach and brain). All images are from 60x original magnification. Scale bars: 20x, 40x and 60x original magnifications correspond respectively to 100μm, 50μm and 30 μm.


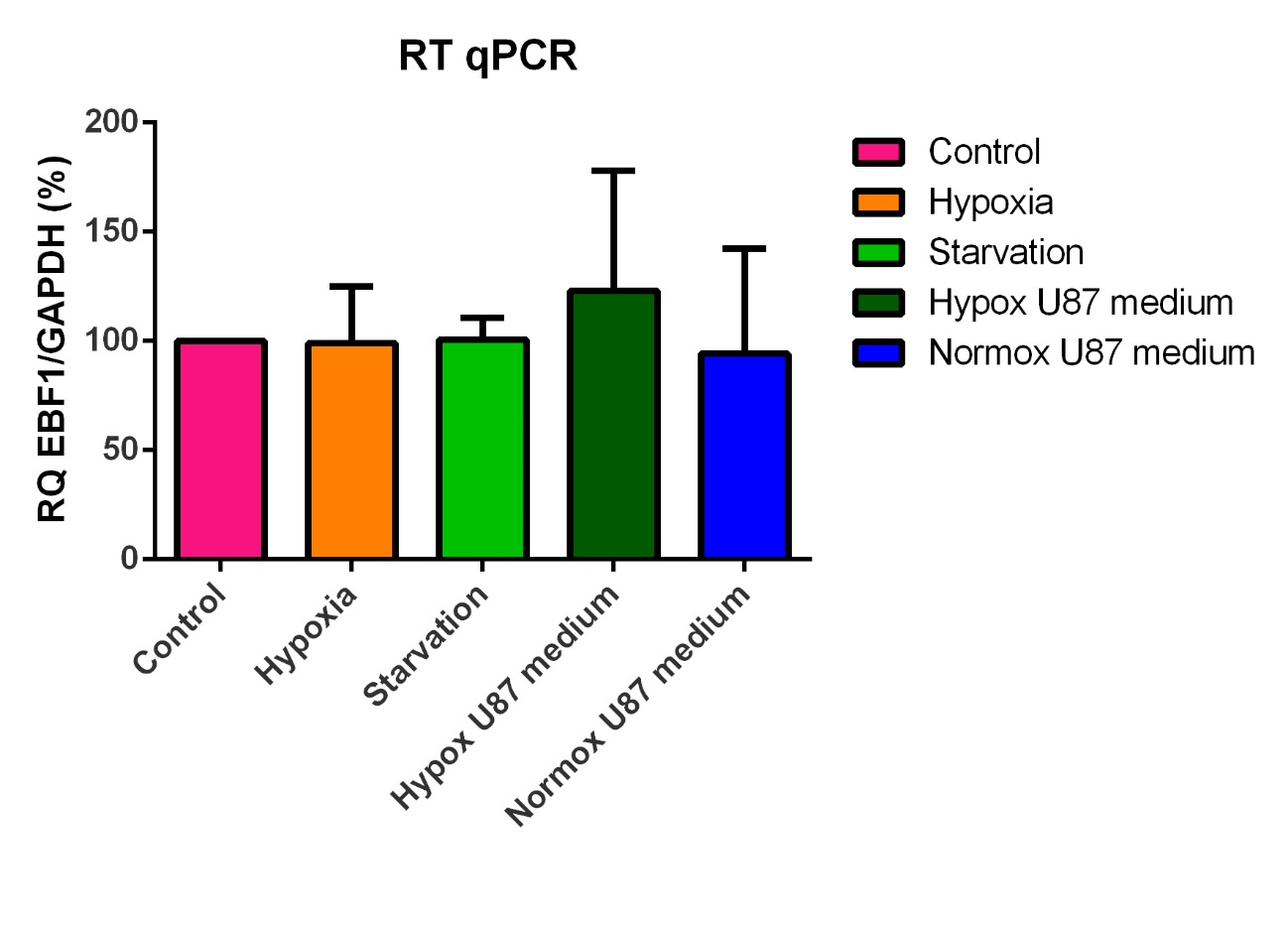


**Supplementary Figure 2. EBF1 expression is not modulated by different culture conditions.**

HBVP were cultured in different conditions, including hypoxia, nutrient starvation and medium obtained from U87 glioblastoma cells exposed to hypoxic and normoxic conditions. The plot shows that EBF1 expression did not change after exposure after 48 hours, if compared to HBVP cultured in standard conditions. The same result was obtained at 24 and 72 hours (not shown), as well as for PL-PC at 24, 48 and 72 hours (not shown). We then performed functional studies downregulating EBF1. Data are presented as mean ± standard deviation of at least 3 independent experiments and one-way analysis of variance with Dunnett's post hoc test were applied.

**Supplementary Figure 3. Silencing of EBF1 does not affect cell proliferation.**

The same number of HBVP cells was plated and cultured at basal conditions or with addiction of the medium obtained from U87 Glioblastoma cells cultured under hypoxic conditions, as a proliferative stimulus. After 48 hours, cells were counted and we could not find any differences in the proliferation rate of silenced cells compared to SCR-treated cells nor at basal or under proliferative stimulus

**SUPPLEMENTARY REFERENCES**

1. Pagani F, Trivedi A, Khatri D*, et al.* Silencing of pantothenate kinase 2 reduces endothelial cell angiogenesis. *Mol Med Reports* 2018; **18**: 4739-4746.

2. De Luca A, Verardi R, Neva A*, et al.* Comparative Analysis of Mesenchymal Stromal Cells Biological Properties. *ISRN Stem Cells* 2013; **2013**: 674671.
